# Supplementary material for: Effects of GABA on Oxidative Stress and Metabolism in High-Glucose Cultured Mongolian Sheep Kidney Cells
Source: Int J Mol Sci. 2024 Sep 18;25(18):10033. doi: 10.3390/ijms251810033 (PMC11432592; doi:10.3390/ijms251810033)
Supplement: Supplementary file 1 [file ijms-25-10033-s001.zip › ijms-3146701-supplementary materials.pdf]

Supplementary Material for

Effects of GABA on Oxidative Stress and Metabolism in High-Glucose Cultured Mongolian Sheep Kidney Cells
This word file includes:

Materials and methods
Tables S1, Tables S2 and Table S3
Results
Tables S4, Tables S5, Tables S6, Tables S7, Tables S8, Tables S9 and Table S10

Materials and methods

4.4 Gene Expression Analysis Using qRT-PCR

Table.S1 Primer sequence of qRT-PCR

Gene Symbol	primer	Annealing temperature (℃)	Product length (bp)
ACTB	F：ATCGGCAATGAGCGGTTC<br>R：TGTTGGCGTAGAGGTCCTT	58	190
CAT	F：AGGCACATGAACGGATATGGAT<br>R：CCTGTGGCGATGGCATTGA	58	188
Gpx1	F：TCCGCTCTTCGCCTTCCTT<br>R：GCTCGATGTCGATGGTCAGAA	58	200
Glut1	F：CCGCTTCCTGCTCATTAACC<br>R：GGCTCTCCTCCTTCATCTCC	58	113
Gpx4	F：CGCAATGAGGCAAGACTGAC<br>R：GAGGACAGGAGTTCTTCAG	58	160
SLC6A1	F：GCCATCAACACCACCAACAT<br>R：GCGGAGAAGTAGACCACCTT	58	197
SOD1	F：GGTTCCACGTCCATCAGTT<br>R：GGTCTCCAACATGCCTCTC	58	120
SOD2	F：GCCATATCAATCACAGCATCTT<br>R：ACCAACAGATACAGCAGTCAG	58	143

Table.S2 T3 column mobile phase gradient condition

time（min）	A（%）	B（%）
10	95	5
7.6	95	5
0	95	5
2	80	20
5	40	60
7.5	1	99
6	1	99

4.6. Chromatography-Mass Spectrometry (CMS) Conditions for Data Acquisition

Table.S3 AB TripleTOF 6600 mass spectrum conditions

Basic parameters	ESI+	ESI-
Duration (min)	10	10
IonSpray Voltage (V)	5000	-4000
Temperature (°C)	550	450
Ion Source Gas1 (psi)	50	50
Ion Source Gas2 (psi)	60	60
Curtain Gas (psi)	35	35
Declustering Potential (V)	60	-60
MS1 Collision Energy (V)	10	-10
MS2 Collision Energy (V)	30	-30
Collision Energy Spread (V)	15	15
MS1 TOF Masses (Da)	50~1000	50~1000
MS2 TOF Masses (Da)	25~1000	25~1000
MS1 Accumulation time (s)	0.2s	0.2s
MS2 Accumulation time (s)	0.04s	0.04s
Candidate ions	18	18
Exclude former target ions	Always, For 3 seconds, After 3 occurences	Always, For 3 seconds, After 3 occurences

Results

2.10. Identification of Differential Metabolites in Renal Cortex Cells

Table.S4 Differential metabolites screened in HG2 vs HG1 group

serial number	Name	VIP	P_{FDR}	FC	Type
1	D - fructose - 6 - phosphate	1.56E+00	2.69E-02	1.87E+00	Up
2	D-glucose 1, 6 diphosphate	1.47E+00	2.88E-02	2.14E+00	Up
3	D-glyceraldehyde-3-phosphate	1.66E+00	1.05E-02	2.07E+00	Up
4	Ribulose - 5 - phosphoric acid	1.49E+00	1.75E-02	2.00E+00	Up
5	D-Su-isocitrate	1.61E+00	2.49E-02	1.92E+00	Up
6	L-glutamic acid	1.62E+00	3.41E-02	5.74E-01	Down

Table.S5 Differential metabolites screened in LG2 vs HG2 group

serial number	Name	VIP	P_{FDR}	FC	Type
1	Citric acid	1.67E+00	2.15E-02	5.84E-01	Up
2	Isocitrate	1.72E+00	1.55E-02	6.75E-01	Up
3	L-glutamic acid	1.72E+00	3.06E-02	1.60E+00	Down
4	D-glucose 1, 6-diphosphate	1.36E+00	2.84E-02	3.43E-01	Up

Table.S6 Heterometabolites screened from LG2 vs LG1 difference group

serial number	Name	VIP	P_{FDR}	FC	Type
1	D-glucose-6-phosphate	1.87E+00	2.53E-02	2.09E+00	Up

2.11. Identification of Differential Metabolites in Renal Medullary Cells

Table.S7 Differential metabolites screened in MHG2 vs MHG1 group

serial number	Name	VIP	P_{FDR}	FC	Type
1	D-xylulose-5-phosphate	1.54E+00	1.61E-02	6.13E-01	Down

Table.S8 Differential metabolites screened in MLG1 vs MHG1 group

serial number	Name	VIP	P_{FDR}	FC	Type
1	glucose	1.54E+00	5.42E-03	2.34E-01	Down

Table.S9 Differential metabolites screened in MLG2 vs MHG2 group

serial number	Name	VIP	P_{FDR}	FC	Type
1	glucose	1.56E+00	1.13E-04	3.50E-01	Up
2	D-glucose-6-phosphate	1.37E+00	3.61E-02	7.87E-01	Up
3	6 - phosphate glucose acid	1.52E+00	3.36E-03	6.87E-01	Up
4	Dihydroxyacetone phosphate	1.51E+00	2.26E-02	3.12E+00	Down

Table.S10 Differential metabolites screened in MLG1 vs MHG1 group

serial number	Name	VIP	P_{FDR}	FC	Type
1	Glutathione is reduced to its original form	1.75E+00	4.62E-02	1.73E+00	Up
2	L-tyrosine	1.82E+00	1.16E-02	1.67E+00	Up
